# Correlation between radiographic measures of acetabular morphology with 3D femoral head coverage in patients with acetabular retroversion

**DOI:** 10.3109/17453674.2012.684138

**Published:** 2012-06-04

**Authors:** Benjamin J Hansen, Michael D Harris, Lucas A Anderson, Christopher L Peters, Jeffrey A Weiss, Andrew E Anderson

**Affiliations:** ^1^Department of Orthopaedics, University of Utah; ^2^Department of Bioengineering, University of Utah; ^3^Department of Physical Therapy, University of Utah, Salt Lake City, UT, USA

## Abstract

**Background and purpose:**

Acetabular retroversion may result in anterior acetabular over-coverage and posterior deficiency. It is unclear how standard radiographic measures of retroversion relate to measurements from 3D models, generated from volumetric CT data. We sought to: (1) compare 2D radiographic measurements between patients with acetabular retroversion and normal control subjects, (2) compare 3D measurements of total and regional femoral head coverage between patients and controls, and (3) quantify relationships between radiographic measurements of acetabular retroversion to total and regional coverage of the femoral head.

**Patients and methods:**

For 16 patients and 18 controls we measured the extrusion index, crossover ratio, acetabular angle, acetabular index, lateral center edge angle, and a new measurement termed the “posterior wall distance”. 3D femoral coverage was determined from volumetric CT data using objectively defined acetabular rim projections, head-neck junctions, and 4 anatomic regions. For radiographic measurements, intra-observer and inter-observer reliabilities were evaluated and associations between 2D radiographic and 3D model-based measures were determined.

**Results:**

Compared to control subjects, patients with acetabular retroversion had a negative posterior wall distance, increased extrusion index, and smaller lateral center edge angle. Differences in the acetabular index between groups approached statistical significance. The acetabular angle was similar between groups. Acetabular retroversion was associated with a slight but statistically significant increase in anterior acetabular coverage, especially in the anterolateral region. Retroverted hips had substantially less posterior coverage, especially in the posterolateral region.

**Interpretation:**

We found that a number of 2D radiographic measures of acetabular morphology were correlated with 3D model-based measures of total and regional femoral head coverage. These correlations may be used to assist in the diagnosis of retroversion and for preoperative planning.

Acetabular retroversion, a recently described acetabular pathomorphology, is characterized by an acetabulum with excessive tilt in the sagittal plane (Reynolds et al. 1999, Giori and Trousdale 2003, Siebenrock et al. 2003a). Retroversion is believed to result in anterior acetabular over-coverage, which can cause pincer-type femoroacetabular impingement (FAI) with subsequent damage to the anterosuperior labrum and cartilage (Siebenrock et al. 2003b, Banks and Grayson 2007, Tannast et al. 2008). Retroversion is associated with posterior acetabular deficiency (Fujii et al. 2010). Cartilaginous lesions to the posteroinferior region of the acetabulum may result from the contrecoup effect, in which anterior impingement causes subluxation and forces the femoral head posteriorly in the acetabulum in a deleterious manner (Beck et al. 2005).

Acetabular retroversion can be difficult to diagnose, and selection of the appropriate treatment strategy presents a challenge. Two-dimensional (2D) radiographs are commonly used to diagnose retroversion and plan treatment (Reynolds et al. 1999, Mast et al. 2004, Jamali et al. 2007, Werner et al. 2010). However, plain radiographs do not quantify the 3-dimensional (3D) relationship between the acetabulum and the femoral head (Clohisy et al. 2009). In addition, radiographic measurements are susceptible to variations in pelvic tilt and poor inter-observer repeatability (Clohisy et al. 2009). 3D models, generated from volumetric CT data, may help to elucidate the magnitude and location of acetabular coverage (Klaue et al. 1988, Dandachli et al. 2009). However, previous studies that have used CT data to estimate coverage have made simplifying assumptions regarding the geometry of the femoral head (Dandachli et al. 2008, 2009), which may have concomitant deformities. Thus, a geometrically accurate comparison between total and regional femoral head coverage in patients with and without acetabular retroversion has not been made.

It is unclear how standard 2D radiographic measures relate to measurements from 3D models. Establishment of correlations between radiographic and model-based measurements of coverage in retroverted hips could guide the interpretation of radiographic findings when it is not feasible to obtain 3D models.

We (1) compared 2D radiographic measurements between patients with acetabular retroversion (patient group) and normal control subjects (control group), (2) compared 3D measurements of total and regional femoral head coverage between the patient and control groups, and (3) quantified statistical relationships between radiographic measurements of acetabular retroversion and 3D measurements of total and regional coverage of the femoral head.

## Patients and methods

### Subjects

The study cohort had 2 groups: a control group (n = 18) consisting of subjects without morphologic hip abnormalities, and a patient group (n = 16) consisting of subjects who presented at our clinic with hip pain and acetabular retroversion. Approval of the University of Utah Institutional Review Board (#28721) was obtained to retrospectively acquire CT image data from control subjects. The control subjects were treated for unilateral pelvic and/or acetabular fractures. Anteroposterior (AP) pelvic radiographs and pelvic CT images were acquired as part of their trauma workup; thus, material from the uninjured hip was available for our evaluation. Control subjects were excluded if they had an inadequate AP radiograph as defined by an obturator index (the ratio of the largest horizontal distance of the obturator foramina on the AP radiograph) of less than 0.8 or greater than 1.2, or if they had a crossover or posterior wall sign (Jacobsen et al. 2004, Tannast et al. 2005). The patients presented at our clinic and were recruited with separate institutional approval, and provided consent (IRB #10983). Acetabular retroversion was diagnosed by the presence of a crossover sign (Reynolds et al. 1999, Jamali et al. 2007).

### 2D radiographic measurements

**Figure 1.**
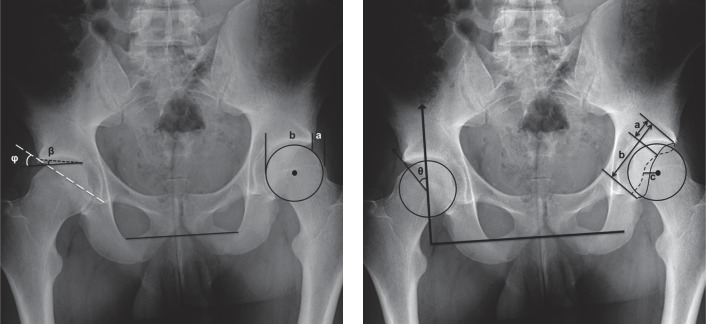
A. Right hip: The *acetabular index* is the angle (β) between the line parallel with pelvic tilt (solid black line which marks the horizontal reference) and the black dashed line from the medial sourcil (sclerotic radiographic density corresponding to the acetabular roof) to the lateral sourcil (where the sourcil meets the lateral acetabular rim). The *acetabular angle* (ϕ) is the angle made by the solid black line and the white dashed line from the acetabular teardrop to the lateral sourcil. Left hip: The *extrusion* index is the amount of femoral head uncovered by the acetabulum (distance a) divided by the diameter of the femoral head (distance a + distance b). B. Right hip: The *lateral center edge angle* (θ) is formed by a line passing through the center of the femoral head perpendicular to the inferior aspect of obturator foramina (thick black line) and a line from the center of the femoral head to the lateral aspect of the congruent sourcil (medial to calcified labra and up-sloping sourcil and even with the posterior wall). Left hip: *Crossover sign* is positive on the left, demonstrated by the anterior wall (solid) crossing the posterior wall (dashed). The *crossover* *ratio* is the ratio of the distance from the lateral-most acetabular rim to the point of the crossover (distance a) divided by the acetabular diameter (the distance from the lateral acetabular rim to the teardrop, b). The *posterior wall distance* is the horizontal distance (distance c) measured from the center of the femoral head to the posterior wall. Distances are positive if the posterior wall is lateral to the head center and negative if medial to the head center.

2D measurements were performed on the radiographs of the control and patient groups. Measurements were performed by 2 investigators (BJH and LA) on 2 separate occasions to determine intra- and inter-observer repeatability. Measurements were performed with tools available in the PACS operating system (Philips iSite PACS v3.6; Philips Healthcare, Andover, MA). The following radiographic measurements were analyzed (Figure 1): acetabular index (AI) (Tönnis 1987, Tannast et al. 2007), acetabular angle (AA) (Sharp 1961), extrusion index (EI) (Li and Ganz 2003, Tannast et al. 2007), lateral center edge angle (LCEA) (Wiberg 1953), crossover ratio (Werner et al. 2008), and a new measurement termed the “posterior wall distance”. The posterior wall distance was measured from the AP pelvis radiograph as the horizontal distance from the center of the femoral head to the posterior wall. Distances were positive if the posterior wall was lateral to the center of the head and negative if it was medial to the center.

### 3D models and measurements

**Figure 2.**
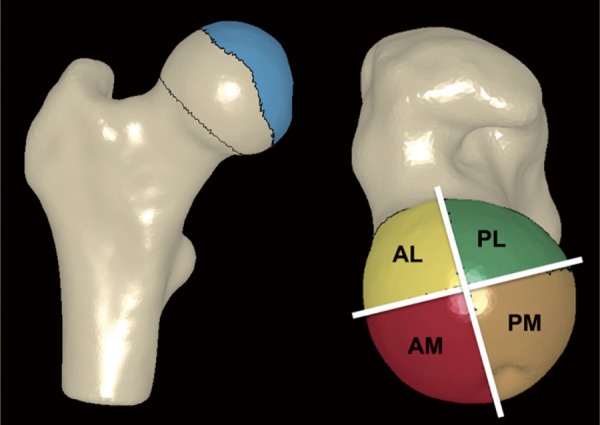
3D reconstruction of femur from CT image data from a control subject. Left: The femur head-neck junction was defined automatically (line at head-neck junction). The region of femoral head that was covered (blue) was determined by projecting the rim of the acetabulum to the femur (line representing boundary of covered region in blue). Right: Two planes were created at the center of the femoral head (white) to divide the head into four anatomical regions. A = anterior, P = posterior, M = medial, and L = lateral. Each region includes the portion of the head from the most superior aspect to the femoral head/neck junction inferiorly.

Volumetric multidetector CT scan images of the entire pelvis were resampled to 1.0-mm-thick axial slices (transverse plane) for each subject. Surfaces of the femoral and pelvic cortical bone were reconstructed semi-automatically using Amira (v5.2.1; Visage Imaging, San Diego, CA) as described previously (Anderson et al. 2005). To measure femoral coverage, a cubic spline was fit to the rim of the acetabulum and projected to the nearest points on the surface of the femoral head to create a line of acetabular coverage (Figure 2). The femoral head-neck junction was defined automatically by first creating a contour map of principal curvatures across the entire femur, and then connecting nodal points of inflection (curvature = 0) circumferentially around the head to form a line (Figure 2). A plane was fit to the inflection points and the head was cut along this plane. Next, the femoral head was divided into anatomic regions by creating a plane based on 3 points: (1) the geometric center of the head when fitted to a sphere, (2) the center of the narrowest cross-section of the neck, and (3) the circumferential center of the femoral shaft. A second plane was then created perpendicular to the first. The bisecting planes defined 4 anatomical regions (Figure 2): anterolateral (AL), anteromedial (AM), posterolateral (PL), and posteromedial (PM). These regions included the entire femoral head from the most superior aspect of the head to the head/neck junction inferiorly in each respective region. The anterolateral and anteromedial regions were combined to define total anterior surface area and the posterolateral and posteromedial were combined to define the total posterior surface area. Using the line of acetabular coverage and the regionalized femoral head, the percent coverage of each region was determined. Coverage areas were calculated as a percent of the total region surface area: 100 × [covered area (mm^2^) / total area of region (mm^2^)].

### Statistics

Radiographic measurements from each read and from each observer were averaged to a single value. Descriptive statistics for 2D radiographic measures were calculated using the average value. Student's t-test was used to determine whether there were significant differences between subject groups for 2D radiographic measurements and 3D model-based measurements. For the radiographic measurements, intra-observer and inter-observer reliabilities were evaluated using the intraclass correlation coefficient (ICC). Observer agreement was interpreted as: slight if the ICC < 0.20, fair if 0.21–0.40, moderate if 0.41–0.60, substantial if 0.61–0.80, and almost perfect if > 0.80 (Landis and Koch 1977). Linear regression was used to determine associations between radiographic and 3D model-based measures (with p < 0.05 being significant). For linear regression analysis, independent variables were defined as the 2D measures (e.g. extrusion index). Dependent variables were defined as the 3D model-based measures: total coverage (TC), anterior coverage (Ant), posterior coverage (Post), anterolateral coverage (ALC), anteromedial coverage (AMC), posterolateral coverage (PLC), and posteromedial coverage (PMC). For the regressions, radiographic measurements between and within observers were averaged and data from the control and patient groups were combined as a single dataset for regression. The strength of correlation was assessed with the Pearson correlation coefficient (r). SPSS software version 11.5 was used to calculate descriptive statistics, t-tests, and ICCs. Linear regressions were performed with Stata IC 11 (StataCorp LP, College Station, TX).

## Results

### Radiographic measurements and repeatability

Statistically significant differences in radiographic measurements between controls and patients with acetabular retroversion were observed for the EI, posterior wall distance, and LCEA (Table 1). Specifically, the EI in control subjects (0.17, SD 0.03) was significantly less than in patients (0.21, SD 0.06) (p = 0.02). The control subjects had a positive posterior wall distance (3.40 mm, SD 2.6) whereas the distance was negative for patients (–5.9 mm, SD 3.9) (p < 0.001). The LCEA in patients (26°, SD 6.2) was significantly less than in the control group (31°, SD 4.4) (p = 0.02). There were small differences between groups in the AI (p = 0.1). Specifically, the AI was reduced in controls compared to the retroversion patients. There was essentially no difference in AA. The crossover ratio for patients was (0.08, SD 0.15) (not quantified/compared for the control group as no crossover signs were observed).

**Table 1.** Descriptive statistics for 2D measurements (average of both readers and both reads). Comparison between controls and patients regarding crossover ratio did not apply as there were no control subjects with a crossover sign

	Controls		Retroversion		
	mean	SD	mean	SD	p-value
Extrusion index	0.17	0.03	0.21	0.06	0.02
Crossover ratio	N/A	N/A	0.08	0.15	N/A
Acetabular angle (degrees)	40	2.3	40	3.8	0.6
Acetabular index	3.14	3.7	5.7	5.3	0.1
Lateral center-edge angle (degrees)	31	4.4	26	6.2	0.02
Posterior wall distance (mm)	3.40	2.6	–5.9	3.9	< 0.001

10 of the 12 radiographic measurements had almost perfect intra-observer correlation coefficients (ICC > 0.8) (Table 2). Observer 2 had substantial disagreement in the AI between first and second reads for 1 patient, which resulted in a moderate ICC at 0.52. Inter-observer agreement was slightly less than intra-observer agreement (Table 2). Nevertheless, 7 of the 12 radiographic measurements had almost perfect inter-observer correlation coefficients (ICC > 0.8). The inter-observer ICC was moderate for the extrusion index, acetabular angle, and acetabular index.

**Table 2.** Within (intra-) and between (inter-) observer intraclass correlation coefficients. Values for the crossover ratio were based on data for subjects with acetabular retroversion only (there were no control subjects with a crossover sign).

Measure	Intraobserver		Interobserver	
	Reader 1	Reader 2	Read 1	Read 2
Extrusion index	0.87	0.81	0.42	0.40
Crossover ratio	0.99	0.97	0.89	0.91
Acetabular angle (degrees)	0.85	0.78	0.59	0.49
Acetabular index	0.97	0.52	0.87	0.48
Lateral center-edge angle (degrees)	0.91	0.90	0.80	0.81
Posterior wall distance (mm)	0.99	0.98	0.93	0.93

### 3D measurements

**Figure 3.**
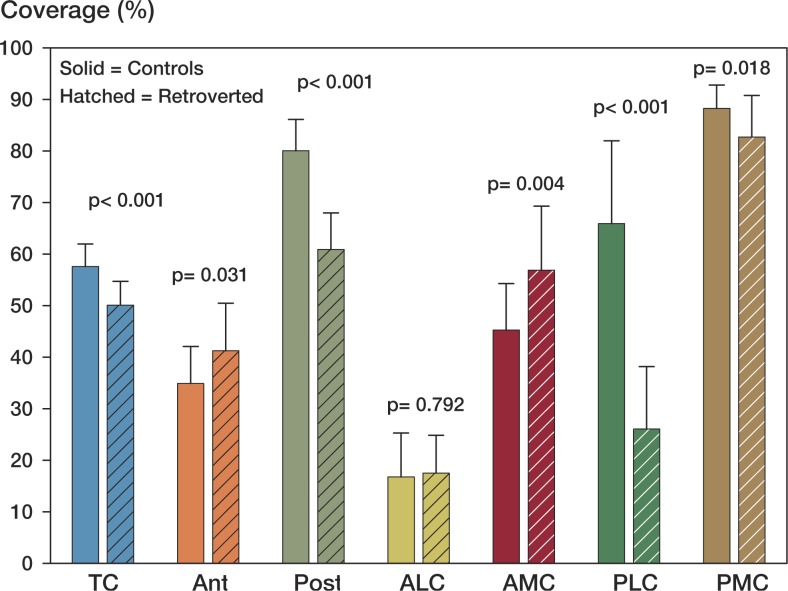
Comparisons of coverage between controls (solid) and subjects with acetabular retroversion (hatched), overall and by regions. Error bars represent SD. TC: total coverage; Ant: anterior coverage; Post: posterior coverage; ALC: anterolateral coverage; AMC: anteromedial coverage; PLC: posterolateral coverage; PMC: posteromedial coverage.

Patients had significantly less total coverage (50%, SD 4.6) than control subjects (58%, SD 4.4) (p < 0.001) (Figure 3). Patients also had slightly, but significantly greater anterior coverage (41%, SD 9.2) than the control subjects (35%, SD 7.2) (p = 0.03). Posterior coverage in patients was substantially and significantly less (61%, SD 7.1) than in control subjects (80%, SD 6.1) (p < 0.001). There were significant differences in AMC, PLC, and PMC between groups (Figure 3). Specifically, AMC was greater in the patient group whereas PLC and PMC were reduced. The greatest difference between groups was for PLC, where coverage for the patients (26%, SD 12.) was substantially less than for the controls (66%, SD 16). There were no significant differences in ALC between groups.

### Regression

**Figure 4.**
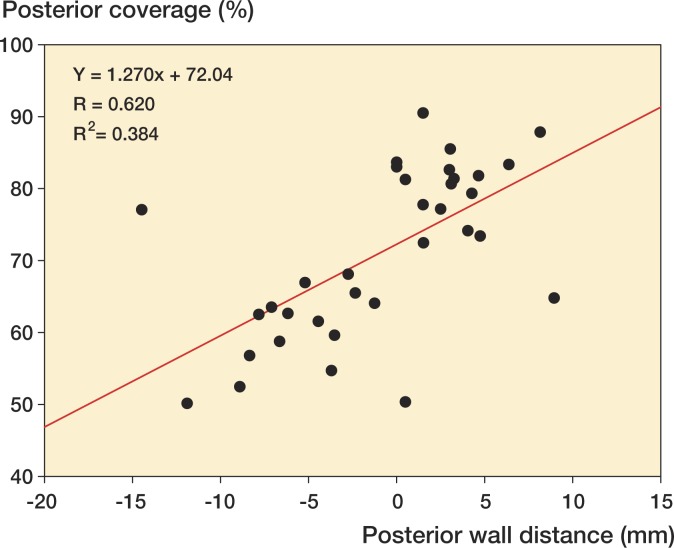
Scatter plot of posterior wall distance against posterior coverage. The solid bar represents regression line.

Regression analysis showed that some 2D measurements were significantly correlated with 3D model-based measurements (Table 3). Specifically, the extrusion index was correlated with TC, Post, and PLC. The posterior wall distance was correlated with TC, Post, PLC, and PMC. The acetabular index was correlated with PLC. Finally, the LCEA was correlated with TC, Post, and PLC. The strongest correlation was between the posterior wall distance and Post (r = 0.62, β = 1.27, p < 0.001) (Figure 4). Regression analysis showed that none of the radiographic measurements correlated significantly with anterior coverage.

**Table 3.** Relationships between 2D radiographic measurements and 3D measurements of coverage.

	TC	Ant	Post	ALC	AMC	PLC	PMC
*3D Measurements*							
Extrusion index	r = –0.37	r = 0.21	r = –0.45	r = –0.021	r = 0.28	r = –0.38	r = –0.30
	β = –42.2	β = 35.7	β = –103.6	β = –3.20	β = 66.9	β = –182.8	β = –41.3
	p = 0.03 **^a^**	p = 0.2	p = 0.007 **^a^**	p = 0.9	p = 0.1	p = 0.03 **^a^**	p = 0.08
Crossover ratio	r = 0.29	r = 0.075	r = 0.21	r = 0.16	r = 0.005	r = 0.22	r = 0.05
	β = 9.03	β = 4.68	β = 9.99	β = 7.92	β = 0.43	β = 18.49	β = 2.59
	p = 0.2	p = 0.8	p = 0.4	p = 0.5	p = 1.0	p = 0.4	p = 0.9
*2D Measurements*							
Posterior wall distance	r = 0.57	r = –0.18	r = 0.62	r = 0.07	r = –0.29	r = 0.61	r = 0.34
	β = 0.59	β = –0.28	β = 1.27	β = 0.10	β = –0.63	β = 2.64	β = 0.42
	p < 0.001 **^a^**	p = 0.3	p < 0.001 **^a^**	p = 0.6	p = 0.09	p < 0.001 **^a^**	p = 0.05 **^a^**
Acetabular angle	r = –0.013	r = 0.16	r = –0.13	r = –0.037	r = 0.21	r = –0.16	r = 0.04
	β = –0.025	β = 0.46	β = –0.50	β = –0.095	β = 0.83	β = –1.30	β = 0.09
	p = 0.9	p = 0.4	p = 0.5	p = 0.8	p = 0.2	p = 0.3	p = 0.8
Acetabular index	r = –0.21	r = 0.18	r = –0.31	r = –0.055	r = 0.26	r = –0.38	r = 0.024
	β = –0.27	β = 0.34	β = –0.77	β = –0.093	β = 0.69	β = –2.04	β = 0.036
	p = 0.2	p = 0.3	p = 0.08	p = 0.8	p = 0.1	p = 0.03 **^a^**	p = 0.8
Lateral center-edge angle	r = 0.48	r = –0.086	r = 0.47	r = 0.17	r = –0.21	r = 0.44	r = 0.23
	β = 0.49	β = –0.13	β = 0.96	β = 0.23	β = –0.44	β = 1.92	β = 0.28
	p = 0.005 **^a^**	p = 0.6	p = 0.005 **^a^**	p = 0.3	p = 0.2	p = 0.01 **^a^**	p = 0.2

**^a^** Significant relationships TC: total coverage; Ant: anterior coverage; Post: posterior coverage; ALC: anterolateral coverage; AMC: anteromedial coverage; PLC: posterolateral coverage; PMC: posteromedial coverage; r: correlation coefficient; b: regression coefficient (slope).

## Discussion

The 2D radiographic measurements with statistically significant differences between groups were the posterior wall distance, LCEA, and extrusion index. The posterior wall distance had near-perfect intra- and inter-observer agreement. Thus, the posterior wall distance may be used to augment the diagnosis of acetabular retroversion in patients with a crossover sign. To our knowledge, posterior wall distance has not been described previously in the literature. With high intra-observer repeatability, the LCEA may also serve as a supplementary measure. Moderate inter-observer repeatability for the extrusion index suggests that it may also have diagnostic value.

There is some disagreement in the literature as to how retroversion alters coverage. We found that retroverted acetabula provided less total coverage, slight anterior over-coverage, and substantial posterior under-coverage. Using a foam model of the pelvis, Giori and Trousdale (2003) suggested that the crossover sign and retroversion was the result of posterior deficiency alone. Using CT scans of patients with developmental dysplasia (with a subset showing signs of retroversion), Fujii et al. (2010) found that posterior and posterosuperior coverage was reduced in retroverted acetabula, but there were no differences between retroverted and anteverted acetabula with regard to anterior coverage. Dandachli et al. (2009) evaluated the relationship between the crossover sign and acetabular retroversion to percent coverage of the femoral head in the retroverted acetabulum. While they found similar total coverage between normal and retroverted acetabula, they noted anterior over-coverage and posterior under-coverage in the retroverted acetabula.

Previous studies have made various, simplifying assumptions when estimating coverage, which may explain discrepancies with regard to how retroversion alters coverage. For example, Dandachli et al. (2009) assumed that femoral heads were spherical. However, it is well known that femoral head asphericity is common in acetabular retroversion (Beck et al. 2005, Steppacher et al. 2008). In the study by Dandachli et al. (2009), half of the hips with acetabular retroversion also had a cam-type deformity. The method we used made no prior assumptions regarding the geometry of the femoral head. Instead, calculations of coverage were accurately obtained from the native geometry (Anderson et al. 2005). Thus, our approach can account for concomitant deformities of the femoral head when calculating coverage.

While we found that anterior coverage was increased in retroverted acetabula, the amount of over-coverage (∼10%) was much less than the magnitude of posterior coverage loss (∼40%). Retroversion was diagnosed by the presence of the crossover sign alone. Thus, our results indirectly suggest that the crossover sign may be the primary result of deficient posterior coverage rather than excessive anterior coverage. Thus, in patients with a crossover sign as the presenting abnormality, surgery aimed at removing excess anterior wall alone may not be treating the source of the morphologic abnormality. In such cases, acetabular reorientation may effectively normalize coverage in the anterior and posterior regions simultaneously. Dandachli et al. (2009) also tended to support acetabular reorientation as a more appropriate treatment than removing part of the anterior wall alone.

Our findings suggest that the posterior wall distance, extrusion index, acetabular index, and lateral center edge angle may predict total and regional posterior coverage. Specifically, posterior wall distance and LCEA were positively correlated with coverage, while EI and AI were negatively correlated with coverage. The directions of these correlations are logical, as increased posterior wall distance and LCEA indicate greater coverage, whereas increased AI and EI describe reduced coverage.

Posterior deficiency may cause hip pain and early arthritis through increased cartilage contact pressures in the posterior region, which lacks sufficient coverage for support (Ezoe et al. 2006). This is consistent with the work of Bardakos and Villar (2009), who noted that positive posterior wall signs were 1 of 2 factors associated with progression to early osteoarthritis. The posterior wall distance we describe offers a new and simple method for characterization of posterior deficiency, as it correlated with 3D measures of total/regional posterior coverage. In addition, the high intra- and inter-observer correlation coefficients suggest clinical applicability. Finally, correlations between the posterior wall distance and total and posterior coverage were higher than for EI, AI, and LCEA. Thus, the posterior wall distance may be better suited to predicting coverage than these other radiographic measures. We found correlations between the extrusion index and both total coverage and coverage in the posterior and posterolateral regions. This measurement may therefore be useful for prediction of posterior coverage in hips where the posterior wall distance or LCEA appears normal.

Others have studied the relationship between plain radiographic measurements and CT measurements for recognition of acetabular morphology (Dandachli et al. 2008, Werner et al. 2010). Werner et al. investigated the relationship between the crossover ratio and CT-based measurements of retroversion (Werner et al. 2010). They found a relationship between the crossover ratio, measured on plain radiographs, and the CT-based roof edge angle (r = –0.49, p < 0.001) and equatorial edge angle (r = 0.40, p < 0.001). However, their CT-based measurements were 2D angles, and may not fully characterize acetabular coverage like the 3D measurements evaluated in this paper. Thus, interpretation of the crossover ratio in terms of 3D acetabular coverage was not addressed. We found that the crossover ratio did not correlate with femoral head coverage. The conflicting results may be a result of the 2D angles used in the study by Werner et al.

The present study has several limitations. First, the sample size was smaller than in other studies that have used CT-based measurements to assess retroversion (Ezoe et al. 2006, Dandachli et al. 2009, Fujii et al. 2010). However, as mentioned above, previous studies have used simplified analyses to calculate coverage, which may limit their applicability and require greater sample size to detect smaller differences in coverage compared to our approach. An additional limitation of our study is that a comprehensive clinical history was not available for the control hips. Thus, although hips with records or evidence of previous surgery or abnormal radiographic morphology (based on radiographic criteria) were excluded, it is not known whether these hips were asymptomatic. In addition, we did not correct all radiographs to a standard pelvic tilt or rotation. While we acknowledge that specialized software is available to correct for pelvic tilt (Zheng et al. 2007), our study was performed without it to follow the standard diagnostic procedure for patients in our clinic and for the majority of published studies. Finally, retroversion was diagnosed by the presence of the crossover sign alone. Recently, however, Perreira et al. (2011) demonstrated that retroversion involves the acetabulum at all levels and includes the entire pelvic segment containing the acetabulum and ischial spine.

In conclusion, acetabular retroversion was associated with a slight but significant increase in anterior acetabular coverage, especially in the anterolateral region. Retroverted hips had substantially less posterior coverage, especially in the posterolateral region. Our study showed that a number of radiographic measures of acetabular morphology were correlated with femoral head coverage. These relationships might be used cautiously by other clinicians to assist in the diagnosis of retroversion and for the purposes of preoperative planning.
